# Supplementary material for: Clustering of single-cell multi-omics data with a multimodal deep learning method
Source: Nat Commun. 2022 Dec 13;13:7705. doi: 10.1038/s41467-022-35031-9 (PMC9748135; doi:10.1038/s41467-022-35031-9)
Supplement: Supplementary file 1 — Supplementary Information [file 41467_2022_35031_MOESM1_ESM.pdf]

Supplementary Figures

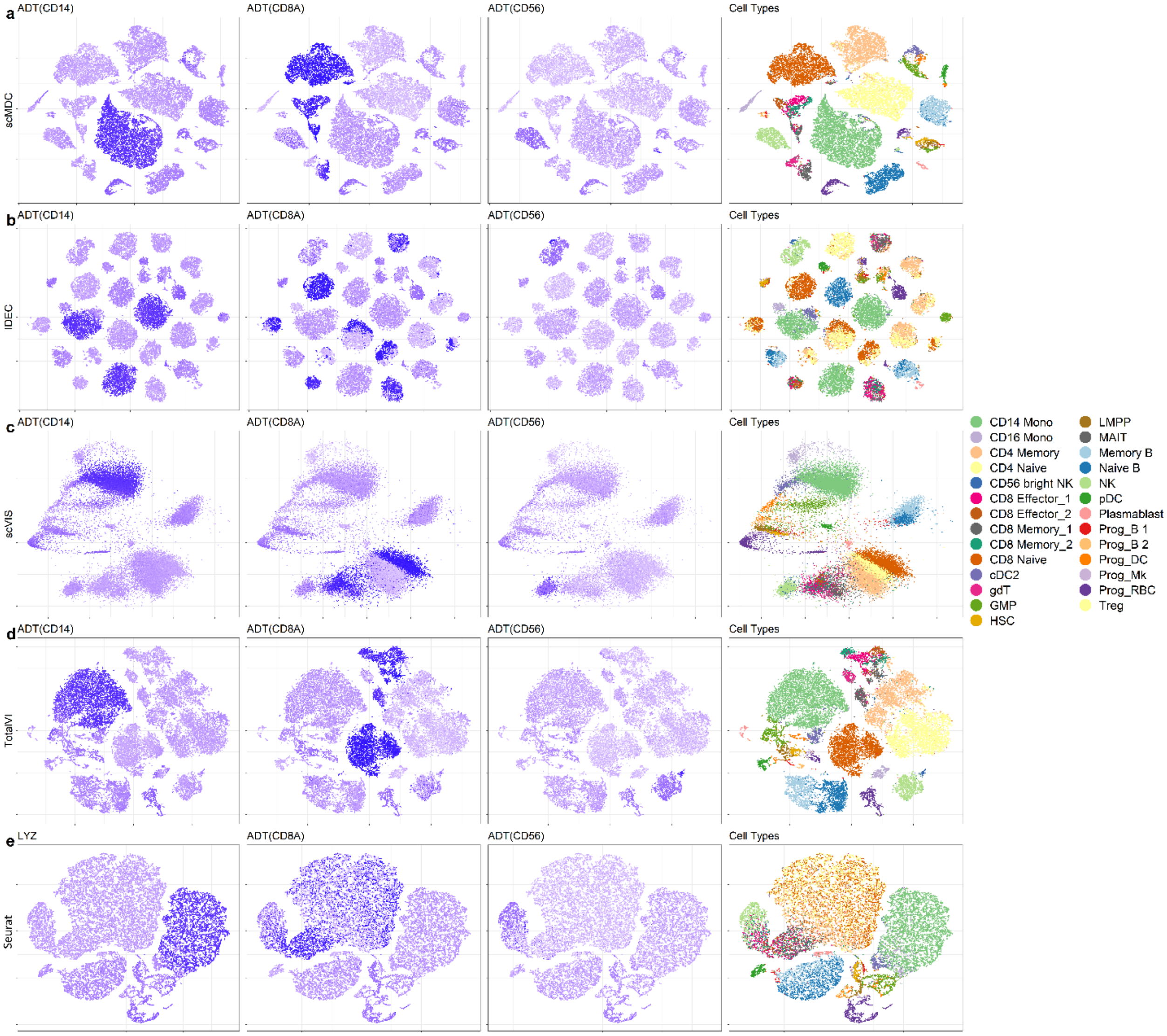

Supplementary Figure 1. Low-dimension representation of scMDC and the competing methods on the BMNC dataset. The t-SNE plots of the embeddings from scMDC (a) and four competing methods including IDEC (b), scVIS (c), TotalVI (d), and Seurat (e) are shown in different rows. The first three columns show the expression pattern of ADT CD14, CD8A, and CD56. The last column shows the true labels (cell types) on the latent space of each method.

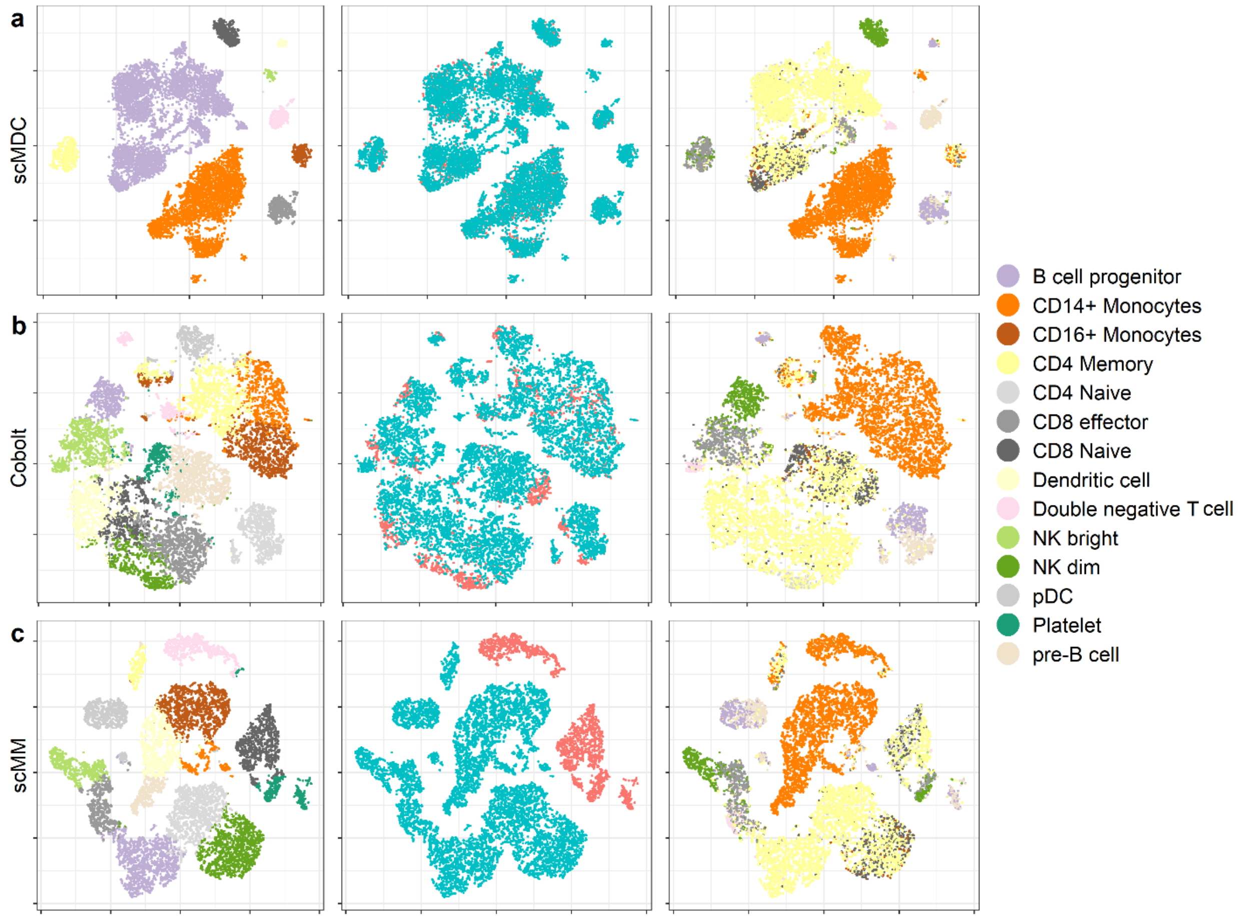

Supplementary Figure 2. Low-dimension representation of scMDC and the competing methods on the PBMC13K dataset. The t-SNE plots of the embeddings from scMDC (a) and two competing methods including Cobolt (b) and scMM (c) are shown in different rows. The three columns show the predicted labels, the batch IDs, and the true labels on the latent space of each method.

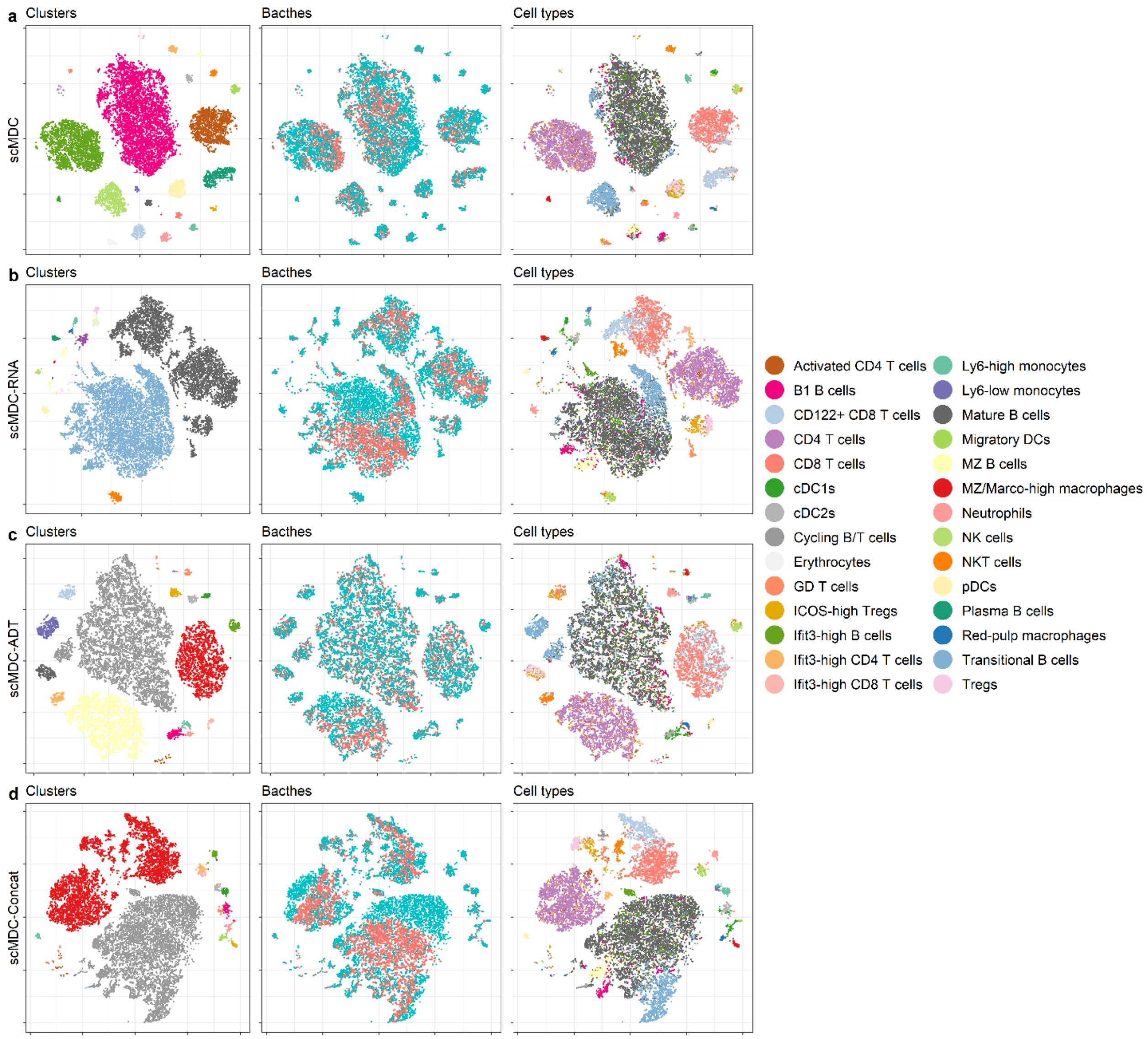

Supplementary Figure 3. Low-dimension representation of scMDC and the variant methods on the SLN111 dataset. The t-SNE plots of the embeddings from scMDC (a) and three competing methods including scMDC-RNA (b), scMDC-ADT (c), and scMDC-Concat (d) are shown in different rows. The three columns show the predicted labels, the batch IDs, and the true labels on the latent space of each method.

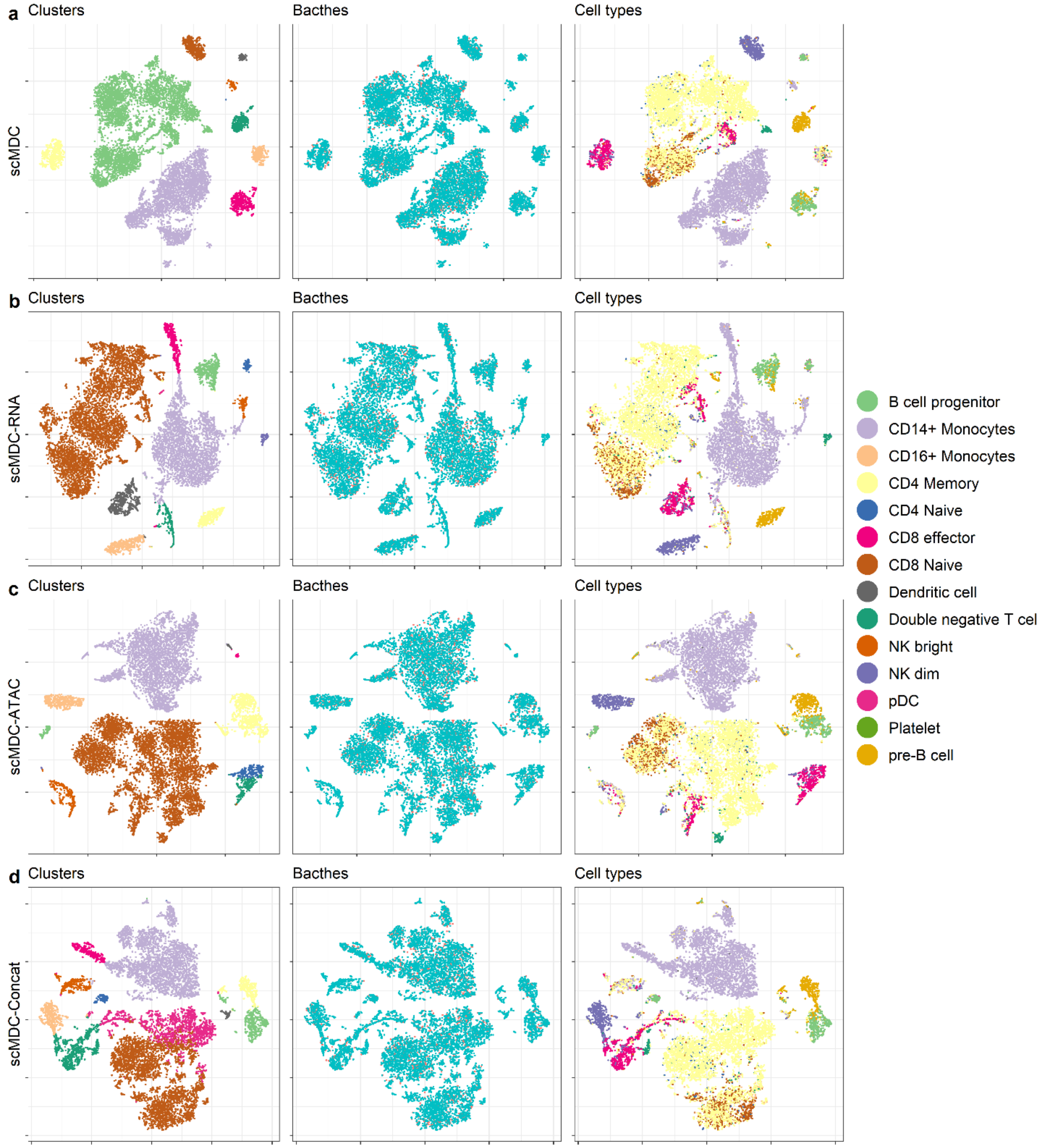

Supplementary Figure 4. Low-dimension representation of scMDC and the variant methods on the PBMC13K dataset. The t-SNE plots of the embeddings from scMDC (a) and three competing methods including scMDC-RNA (b), scMDC-ATAC (c), and scMDC-Concat (d) are shown in different rows. The three columns show the predicted labels, the batch IDs, and the true labels on the latent space of each method.

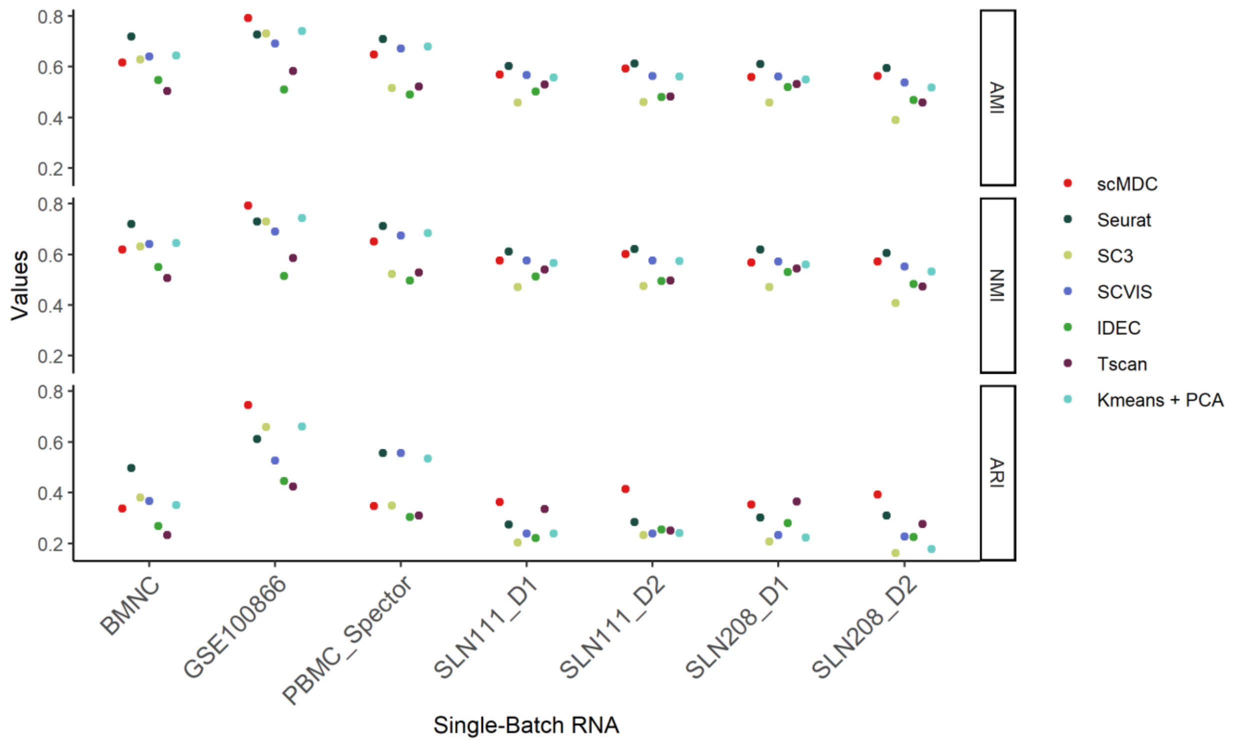

Supplementary Figure 5. Clustering performance of scMDC-RNA and six single-modal clustering methods on the single-batch CITE-seq datasets. All methods only take mRNA counts or normalized counts as input. Clustering performance is evaluated by AMI, NMI, and ARI. Source data are provided as a Source Data file.

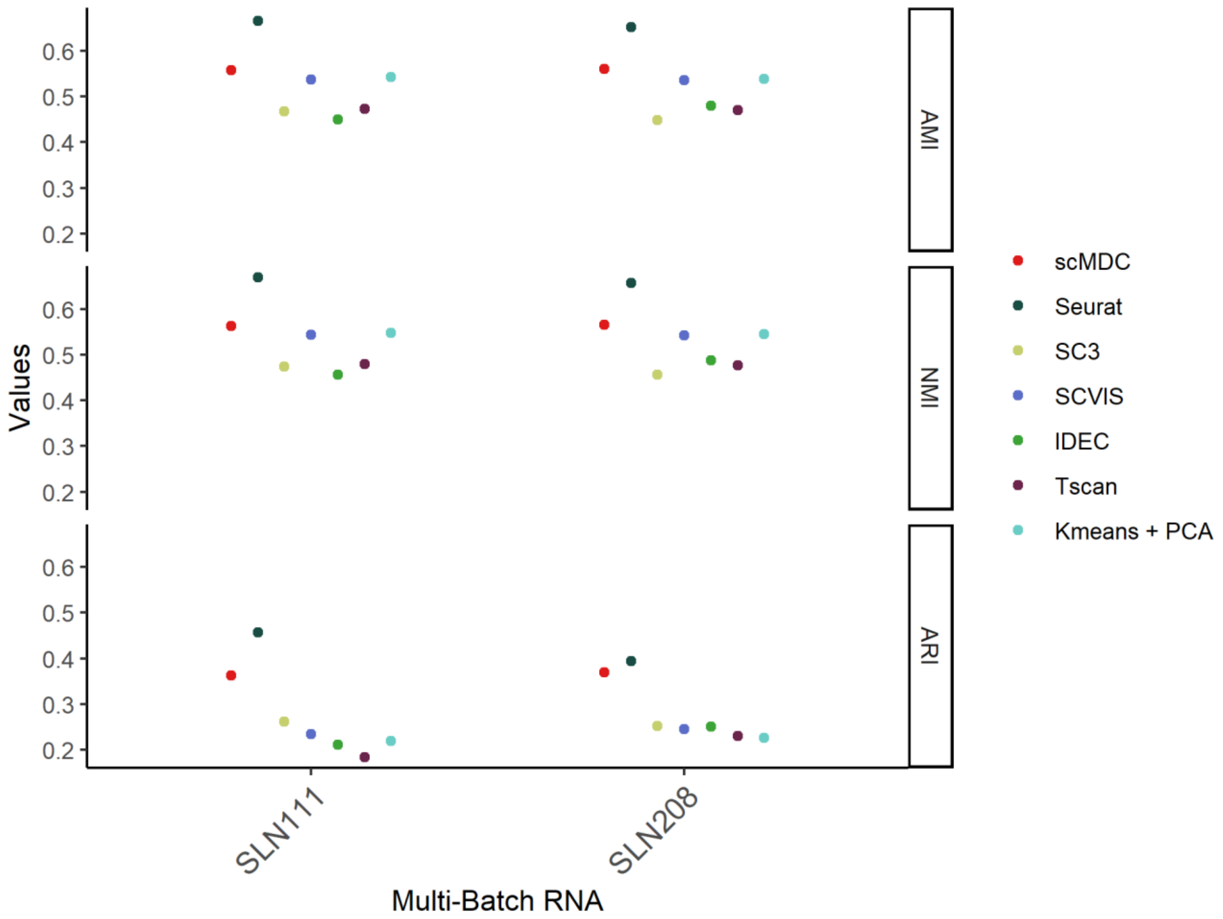

Supplementary Figure 6. Clustering performance of scMDC-RNA and six single-modal clustering methods on the multiple-batch CITE-seq datasets. All methods only take mRNA counts or normalized counts as input. Clustering performance is evaluated by AMI, NMI, and ARI. Source data are provided as a Source Data file.

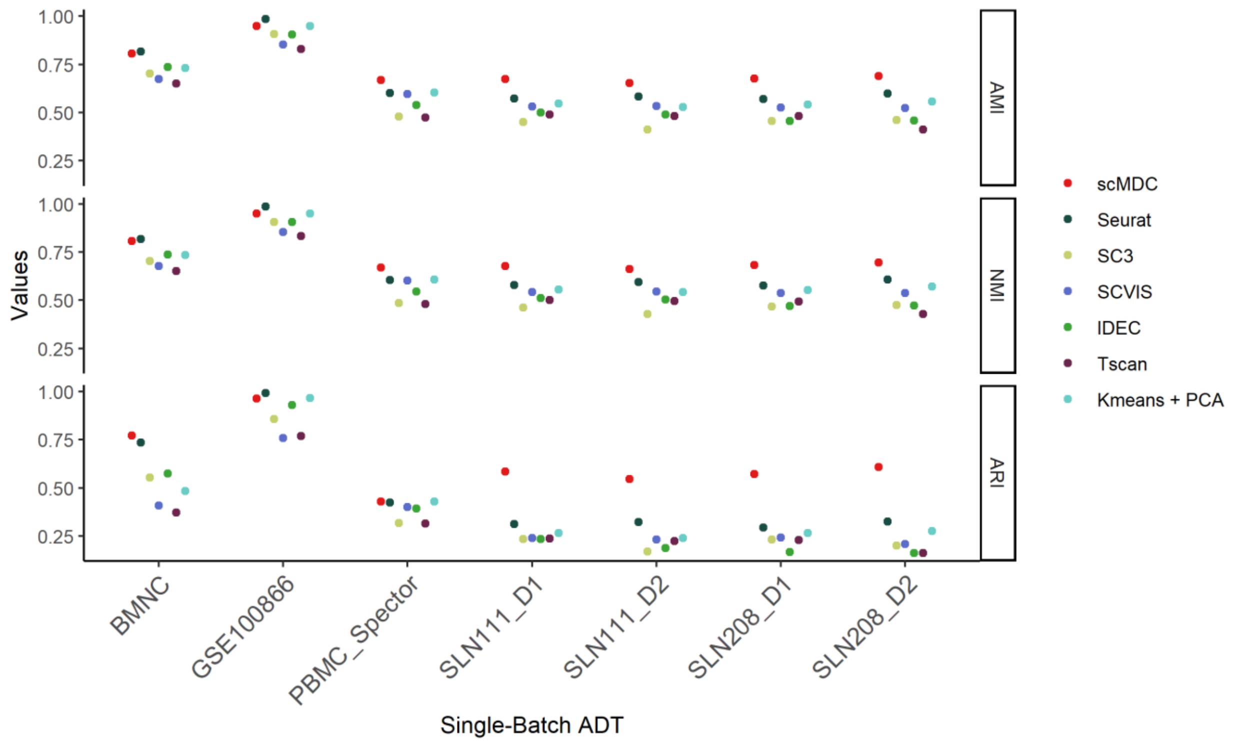

Supplementary Figure 7. Clustering performance of scMDC-ADT and six single-modal clustering methods on the single-batch CITE-seq datasets. All methods only take ADT counts or normalized counts as input. Clustering performance is evaluated by AMI, NMI, and ARI. Source data are provided as a Source Data file.

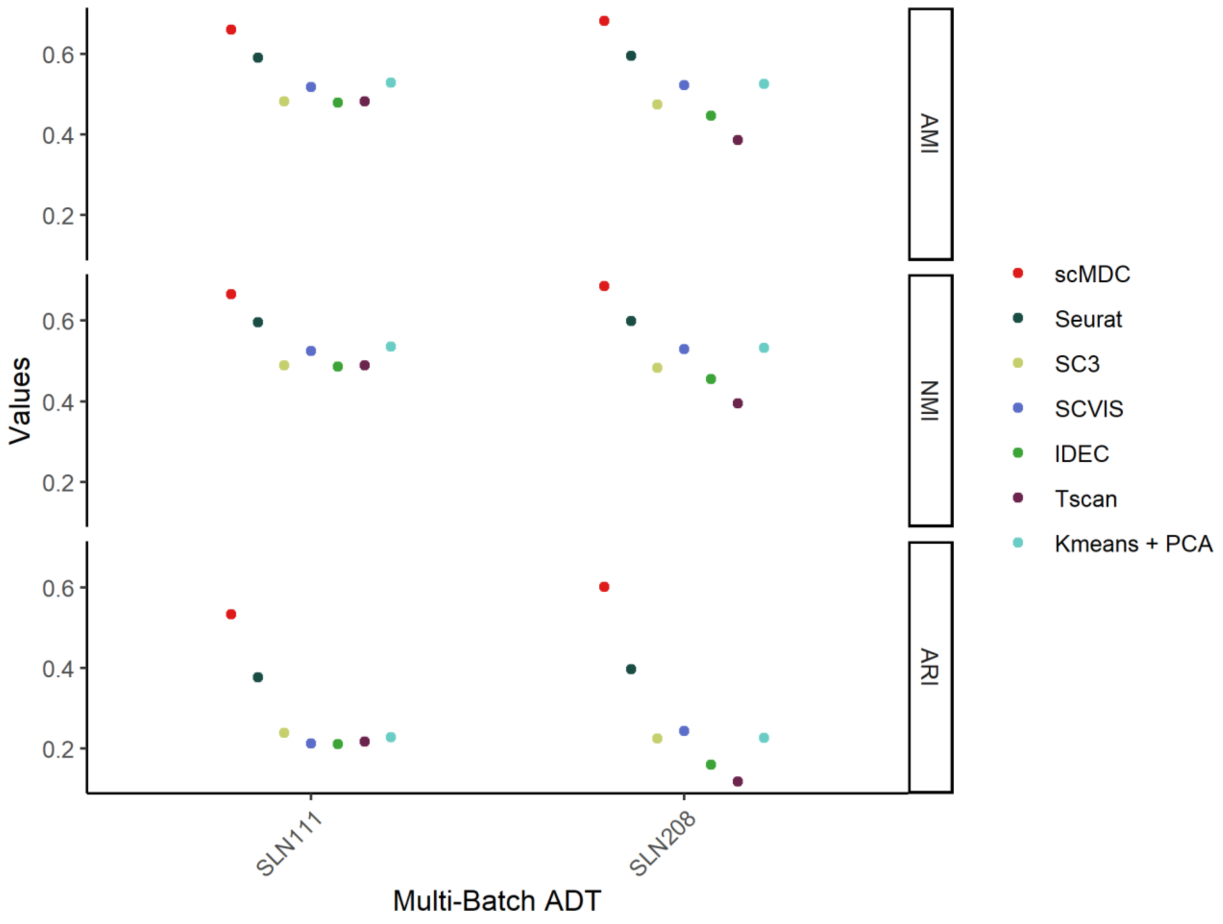

Supplementary Figure 8. Clustering performance of scMDC-ADT and six single-modal clustering methods on the multiple-batch CITE-seq datasets. All methods only take ADT counts or normalized counts as input. Clustering performance is evaluated by AMI, NMI, and ARI. Source data are provided as a Source Data file.

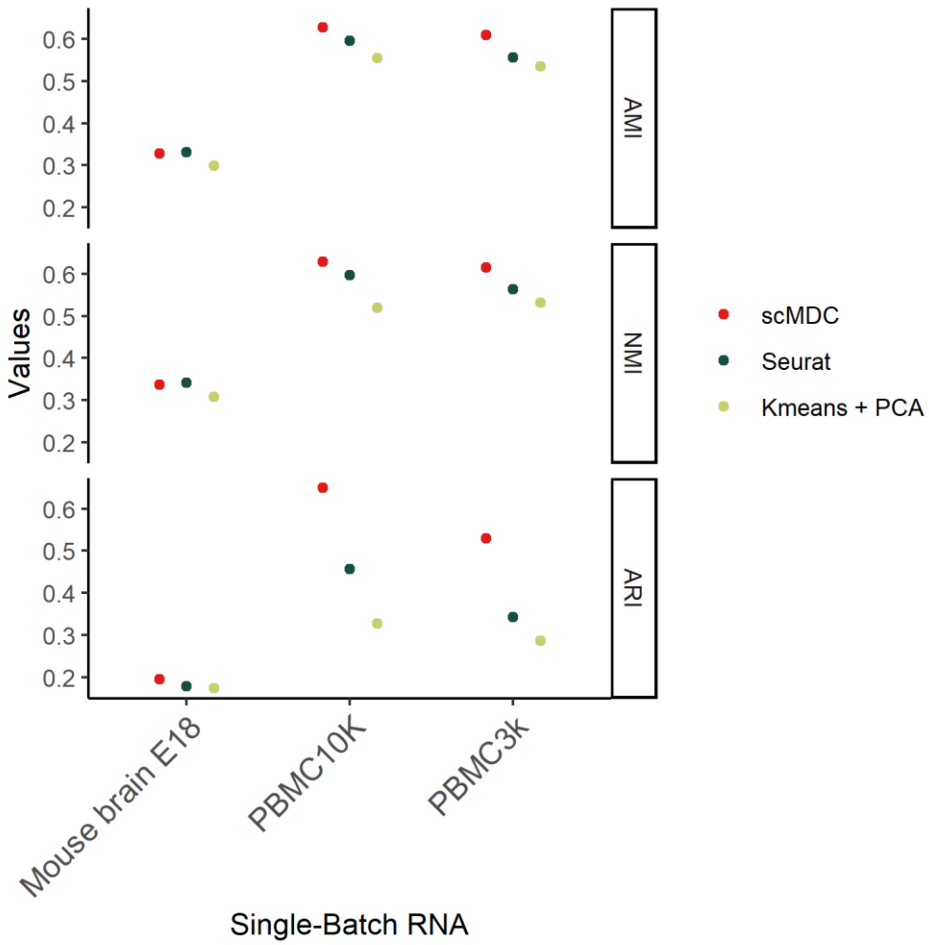

Supplementary Figure 9. Clustering performance of scMDC-RNA and two single-modal clustering methods on the single-batch SMAGE-seq datasets. All methods only take mRNA counts or normalized counts as input. Clustering performance is evaluated by AMI, NMI, and ARI. Source data are provided as a Source Data file.

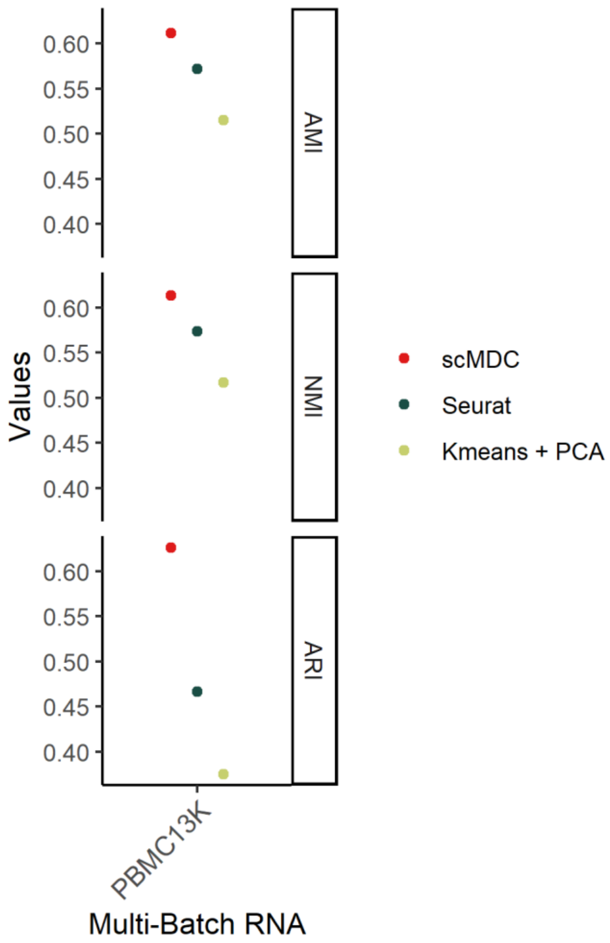

Supplementary Figure 10. Clustering performance of scMDC-RNA and two single-modal clustering methods on a multiple-batch SMAGE-seq dataset. All methods only take mRNA counts or normalized counts as input. Clustering performance is evaluated by AMI, NMI, and ARI. Source data are provided as a Source Data file.

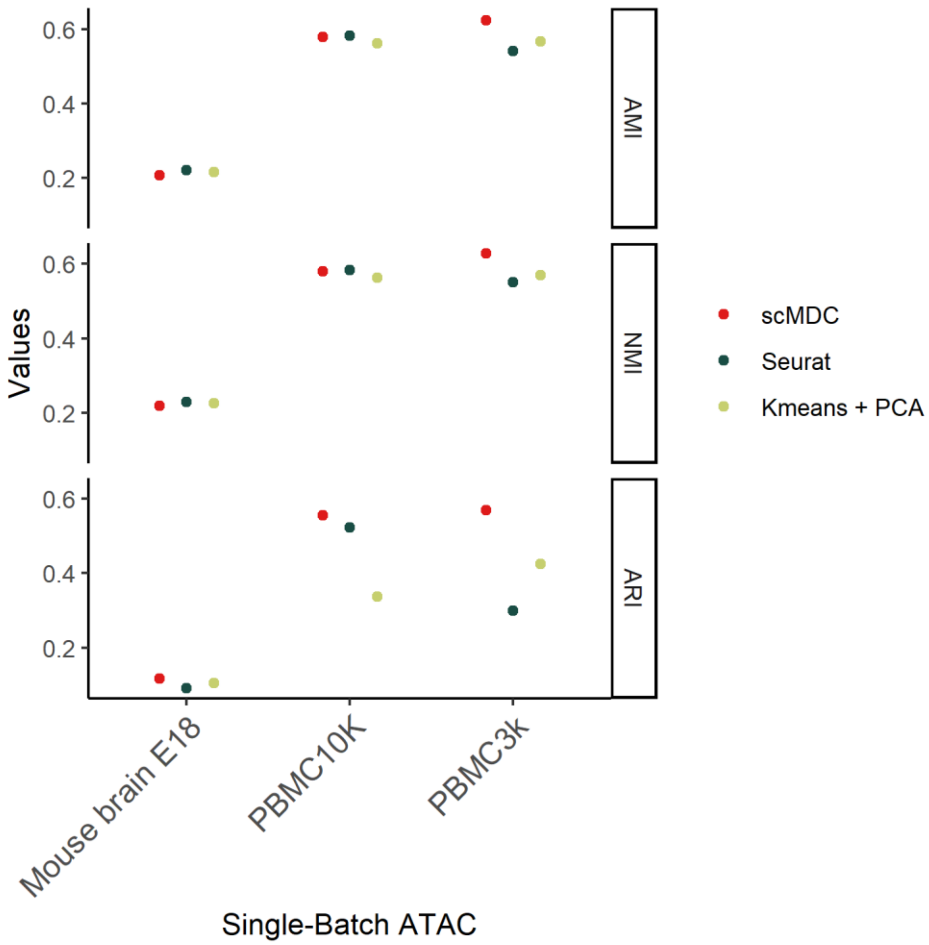

Supplementary Figure 11. Clustering performance of scMDC-ATAC and two single-modal clustering methods on the single-batch SMAGE-seq datasets. All methods only take ATAC counts or normalized counts as input. The ATAC counts are mapped to the gene regions. Clustering performance is evaluated by AMI, NMI, and ARI. Source data are provided as a Source Data file.

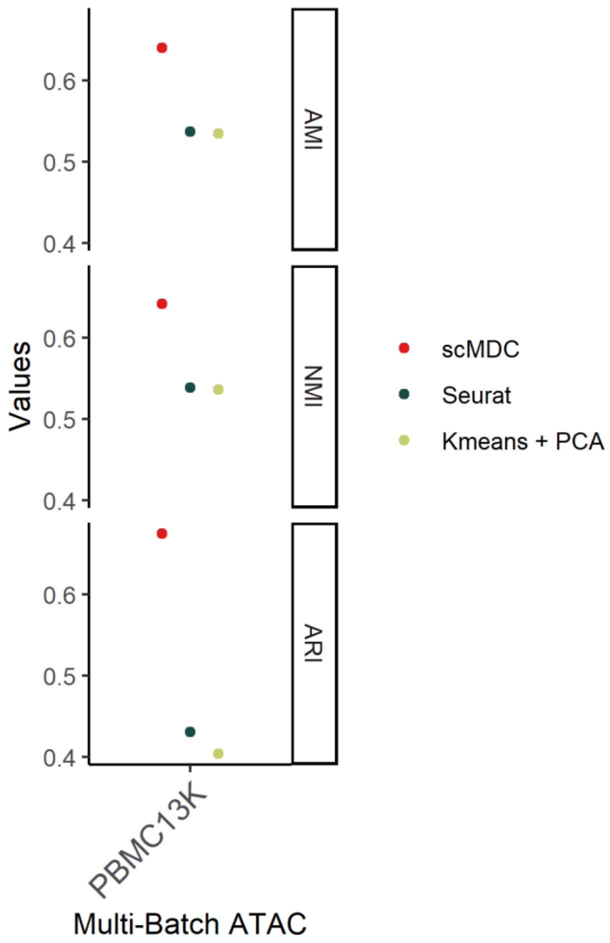

Supplementary Figure 12. Clustering performance of scMDC-ATAC and two single-modal clustering methods on a multiple-batch SMAGE-seq dataset. All methods only take ATAC counts or normalized counts as input. The ATAC counts are mapped to the gene regions. Clustering performance is evaluated by AMI, NMI, and ARI. Source data are provided as a Source Data file.

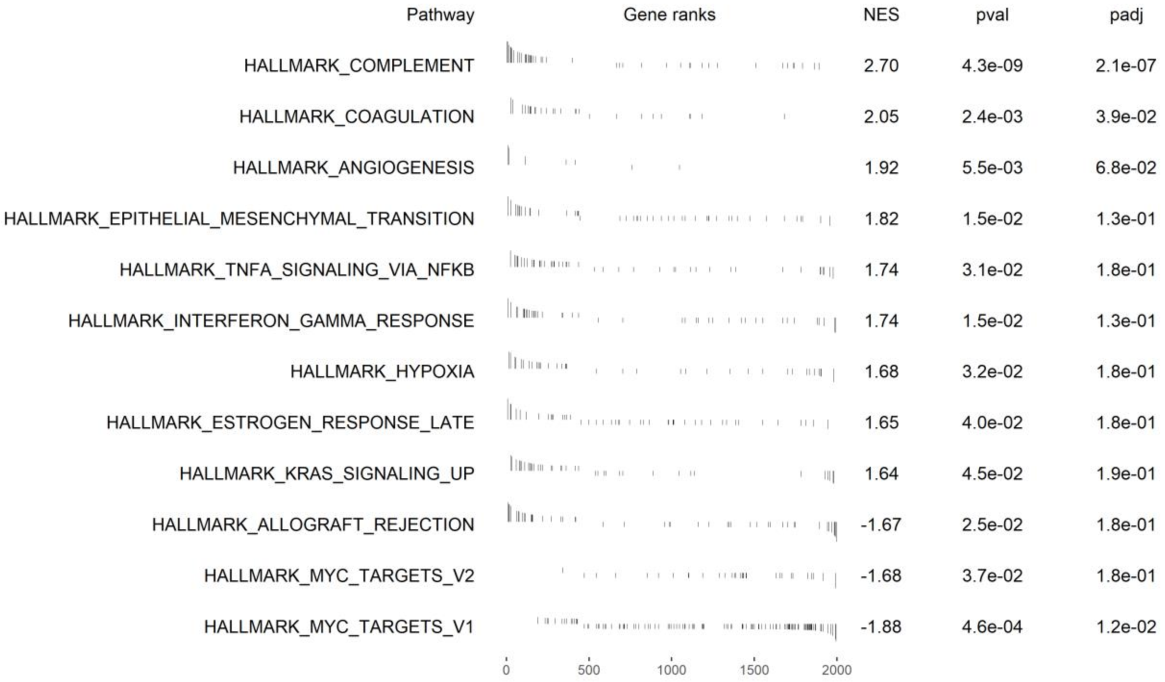

Supplementary Figure 13. Enrichment plot of Hallmark pathways in CD14 monocyte cells from the BMNC dataset. The Kolmogorov-Smirnov test is used here, and the nominal P-values are adjusted for multiple comparisons (padj) by Benjamini & Hochberg (BH) method. Pathways with nominal P-values < 0.05 are shown.

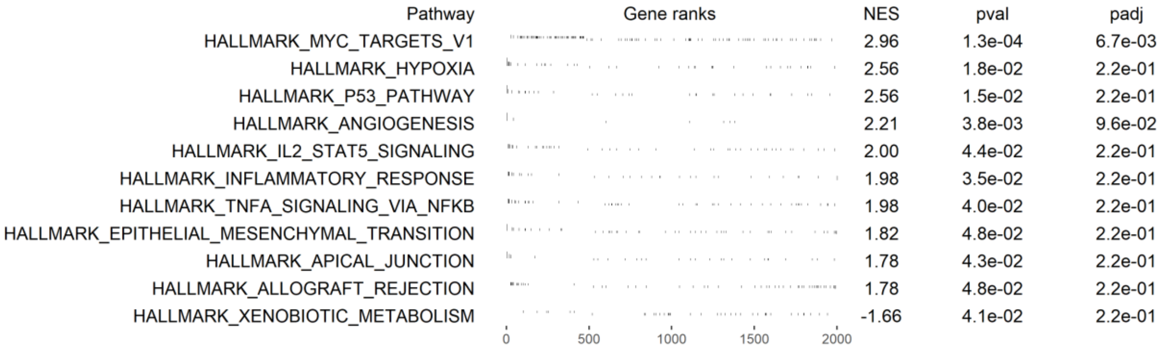

Supplementary Figure 14. Enrichment plot of Hallmark pathways in CD4 memory cells from the BMNC dataset. The Kolmogorov-Smirnov test is used here, and the nominal P-values are adjusted for multiple comparisons (padj) by Benjamini & Hochberg (BH) method. Pathways with nominal P-values < 0.05 are shown.

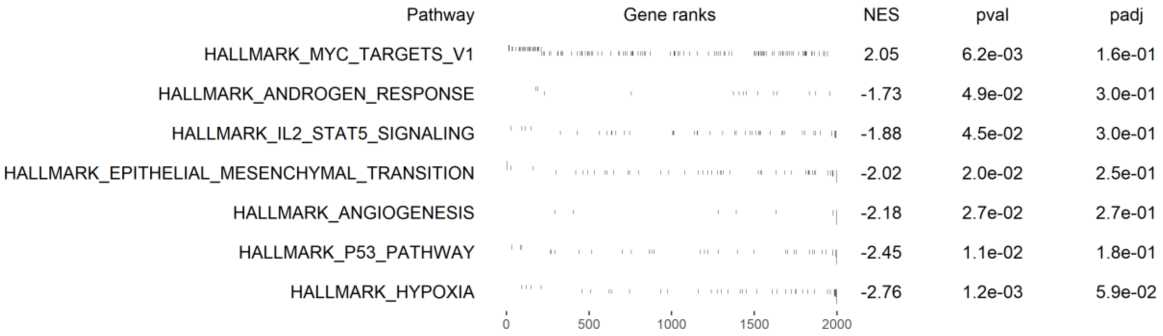

Supplementary Figure 15. Enrichment plot of Hallmark pathways in CD4 naive cells from the BMNC dataset. The Kolmogorov-Smirnov test is used here, and the nominal P-values are adjusted for multiple comparisons (padj) by Benjamini & Hochberg (BH) method. Pathways with nominal P-values < 0.05 are shown.

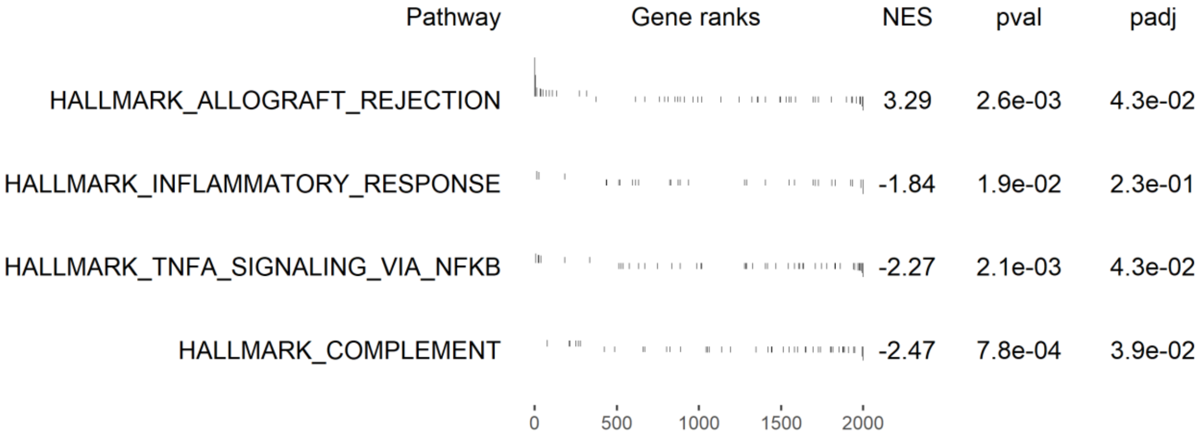

Supplementary Figure 16. Enrichment plot of Hallmark pathways in CD8 naive cells from the BMNC dataset. The Kolmogorov-Smirnov test is used here, and the nominal P-values are adjusted for multiple comparisons (padj) by Benjamini & Hochberg (BH) method. Pathways with nominal P-values < 0.05 are shown.

Supplementary tables

Supplementary Table 1. One-sided paired t-test between the clustering performance of scMDC and the competing methods for the CITE-seq datasets.

Methods	*p_AMI*	*p_NMI*	*p_ARI*
BREM-SC	0.00402824	0.00355908	9.1677E-06
CiteFuse	0.0079801	0.01111861	0.00036261
IDEC	6.7698E-05	7.57E-05	5.7372E-07
Kmeans + PCA	2.1861E-05	2.1894E-05	6.0185E-05
SC3	1.4569E-05	1.366E-05	2.2145E-05
SCVIS	0.00025911	0.00030163	5.887E-06
Seurat	0.00212642	0.00220737	0.00062321
Specter	0.00015003	0.00010859	0.00161893
TotalVI	0.01579666	0.0144765	0.00069109
Tscan	2.0401E-05	2.4565E-05	1.9785E-05

Supplementary Table 2. One-sided paired t-test between the clustering performance of scMDC and the competing methods for the SMAGE-seq datasets.

Methods	*p_AMI*	*p_NMI*	*p_ARI*
Cobolt	0.04201604	0.04327985	0.01847998
Kmeans + PCA	0.00932083	0.00834753	0.01511548
scMM	0.00944043	0.00970153	0.01339524
Seurat	0.01684468	0.01755079	0.01762545

Supplementary Table 3. One-sided paired t-test between the clustering performance of scMDC and the competing methods for the simulation datasets.

Methods	*p_AMI*	*p_NMI*	*p_ARI*
BREMSC	0.00205187	0.00194259	6.9106E-05
CiteFuse	3.9747E-06	3.9025E-06	2.7077E-05
iDEC	5.5039E-07	5.4942E-07	9.7328E-07
PCA+Kmeans	0.00012266	0.00012191	0.00012582
SC3	7.5575E-05	7.5267E-05	9.2593E-06
SCVIS	4.3007E-05	4.2824E-05	9.334E-06
Seurat	5.6212E-06	4.9064E-06	0.00022347
Specter	1.2021E-06	1.4494E-06	1.1547E-05
TotalVI	0.0028567	0.00282413	0.0248134
Tscan	5.0904E-05	5.1844E-05	1.4705E-05

Supplementary Table 4. One-sided paired t-test between the clustering performance of scMDC and the competing methods for the model testing experiments.

Method1	*Method2*	*Pval_AMI*	*Pval_NMI*	*Pval_ARI*
scMDC	ATAC	0.07041006	0.07245205	0.09195784
scMDC	Concat-ATAC	0.00194839	0.00135246	0.0296167
scMDC	RNA	0.00015569	0.00016842	0.00013612
scMDC	ADT	0.00124744	0.0011954	0.00185413
scMDC	Concat-ADT	9.0239E-06	9.9314E-06	8.4946E-06

Supplementary Table 5. One-sided paired t-test between the clustering performance of scMDC and the competing methods for the parameter tunning experiments.

Parameters	*Values*	*pvals_ami*	*pvals_nmi*	*pvals_ari*
Fi	0.0001	0.657522448	0.654359414	0.339330244
Fi	0.001	0.061665126	0.061031154	0.15169793
Fi	0.005	0.185708427	0.183215647	0.065754824
Fi	0.01	0.721740687	0.721638474	0.172244312
Fi	0.1	0.996335282	0.996328807	0.993537274
Fi	1	0.999079993	0.99907693	0.998847524
Gamma	0.01	0.404148719	0.402075548	0.465113431
Gamma	0.1	0.020012276	0.019725304	0.027002903
Gamma	1	0.273661585	0.272609533	0.211856888
Gamma	10	0.505974017	0.505992115	0.565385718
Gamma	100	0.859013414	0.858343271	0.82483211

Supplementary Table 6. Summary of the real CITE-seq datasets*

**Datasets**	**Platform**	**Tissue**	**# of cells**	**# of total genes**	**# of ADTs**	**# of groups**
PBMC	10X	PBMC	3,762	33,538	49	16
GSE100866	10X	CBMN	1,372	33,514	10	6
BMNC	10X	BMNC	30,672	17,009	25	27
SLN111D1	10X	SLN	9,264	13,553	111	35
SLN111D2	10X	SLN	7,564	13,553	111	35
SLN208D1	10X	SLN	8,715	13,553	208	35
SLN208D2	10X	SLN	7,105	13,553	208	35

* We selected top 1000 highly dispersed genes for experiments in all datasets

Supplementary Table 7. Summary of the real Single-cell Multiome ATAC Gene Expression datasets*

**Datasets**	**Platform**	**Tissue**	**# of cells**	**# of total genes**	**# of genes from ATAC**	**# of groups**
PBMC3k	10X	PBMC	2,585	36,601	20,010	14
PBMC10K	10X	PBMC	11,020	36,601	20,010	12
MBE18	10X	Brain	4,780	32,285	21,807	18

* We selected top 2000 highly dispersed genes for experiments in all datasets
